# Vaginal leiomyoma: medical imaging and diagnosis in a resource low tertiary hospital: case report

**DOI:** 10.1186/s12905-020-0883-2

**Published:** 2020-01-21

**Authors:** Thomas Obinchemti Egbe, Fidelia Mbi Kobenge, Junette Arlette Mbengono Metogo, Emmanuella Manka’a Wankie, Paul N. Tolefac, Eugene Belley-Priso

**Affiliations:** 1 Faculty of Health Sciences, University of Buea, P.O. Box 63, Buea, Cameroon; 2 Department of Obstetrics and Gynecology, Douala Referral Hospital, P.O. Box 4856, Douala, Cameroon; 3 Faculty of Medicine and Pharmaceutical Sciences, University of Douala, Douala, Cameroon; 4 Department of Anesthesiology and Reanimation, Douala Referral Hospital, Douala, Cameroon; 5 Department of Radiology, Douala Referral Hospital, Douala, Cameroon; 6 Faculty of Medicine and Biomedical Sciences, University of Yaoundé 1, Yaoundé, Cameroon

**Keywords:** Vaginal leiomyoma, Misdiagnosis, Laparoscopy, Magnetic resonance imaging, And ultrasonography

## Abstract

**Background:**

In the literature under review there are about 300 reported cases of vaginal leiomyomas with none from Cameroon. We report a case of vaginal leiomyoma and highlight the diagnostic challenges faced at the Douala Referral Hospital (DRH), Cameroon.

**Case presentation:**

A 36-year-old G3P3002 sexually active Cameroonian married woman reported dysuria, dyspareunia, cessation of sexual intercourse and offensive smelling vaginal discharge for 6 months and a 3-year history of a vaginal tumour; she was misdiagnosed despite ultrasonography and magnetic resonance imaging (MRI) but was corrected by an experienced radiologist. She underwent first look laparoscopy, surgical excision of the tumour through the vagina and histopathology analysis that confirmed leiomyoma.

**Conclusion:**

Posterior location of vaginal leiomyomas found in this case is a rare occurrence. The diagnosis is based on careful examination and preoperative imaging (ultrasonography and MRI). However, the definitive diagnosis is usually made intra-operatively. We combined laparoscopic exploration of the internal genital organs and per vaginal excision of the vaginal leiomyoma. Thus, we recommend frozen section biopsy to exclude leiomyosarcoma.

## Background

Vaginal leiomyomas are rare benign tumours with only about 300 reported cases in medical literature [1]. To the best of our knowledge, there has been no case of vaginal leiomyoma reported in the medical literature from sub-Saharan Africa, and Cameroon in particular. These tumours usually arise from the anterior vaginal wall and, depending on the size and site, may cause varied clinical presentations such as dyspareunia, pain, or dysuria [2]. Vaginal leiomyomas sometimes occur concurrently with leiomyomas elsewhere in the body [3]. The clinical diagnosis of vaginal leiomyoma requires a high index of suspicion because the tumour could easily be mistaken for a cystocele, urethrocele, Skene duct abscess, Gartner duct cysts, urethral diverticulum, vaginal cysts, Bartholin gland cysts, or vaginal malignancy [4, 5]. The diagnosis is usually confirmed by histopathology.

We are reporting the case of a vaginal leiomyoma that we treated with first look laparoscopy and surgical excision through the vaginal route at the Douala Referral Hospital (DRH), Cameroon.

## Case presentation

**Fig. 1:**
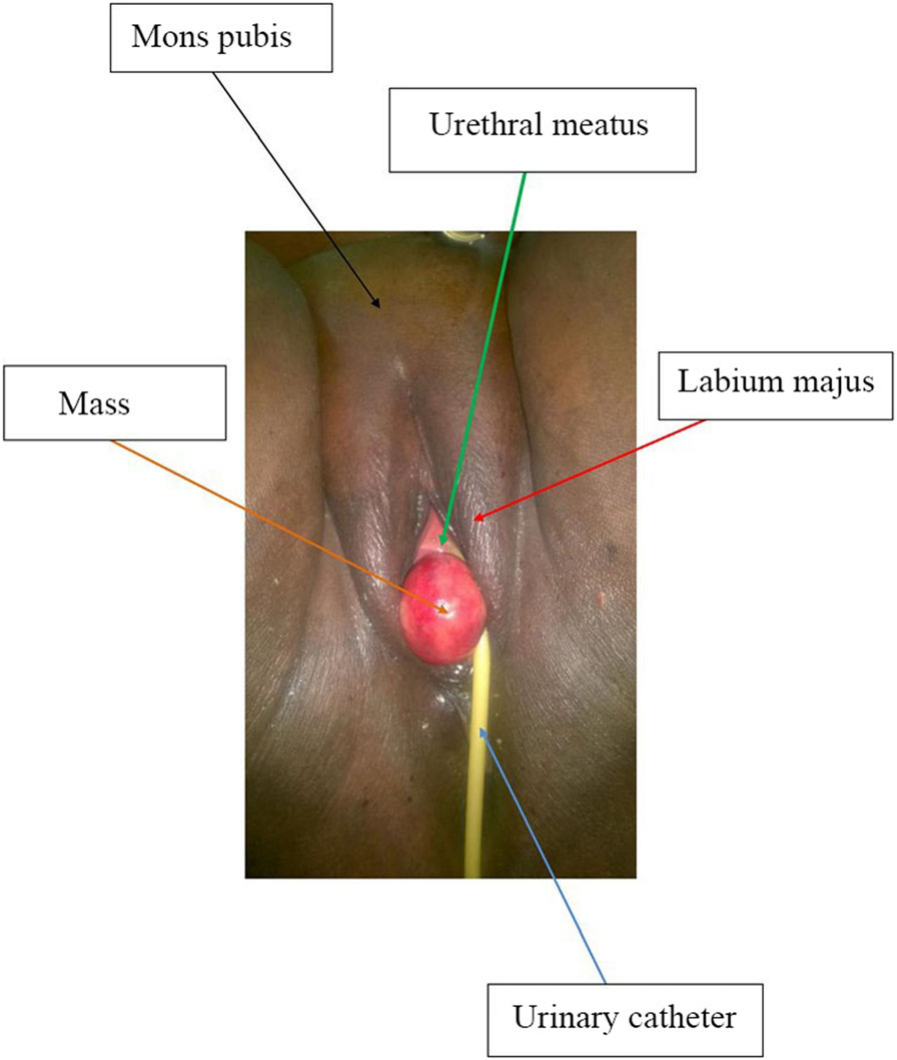
Tumour occluding and projecting through the vagina

**Fig. 2:**
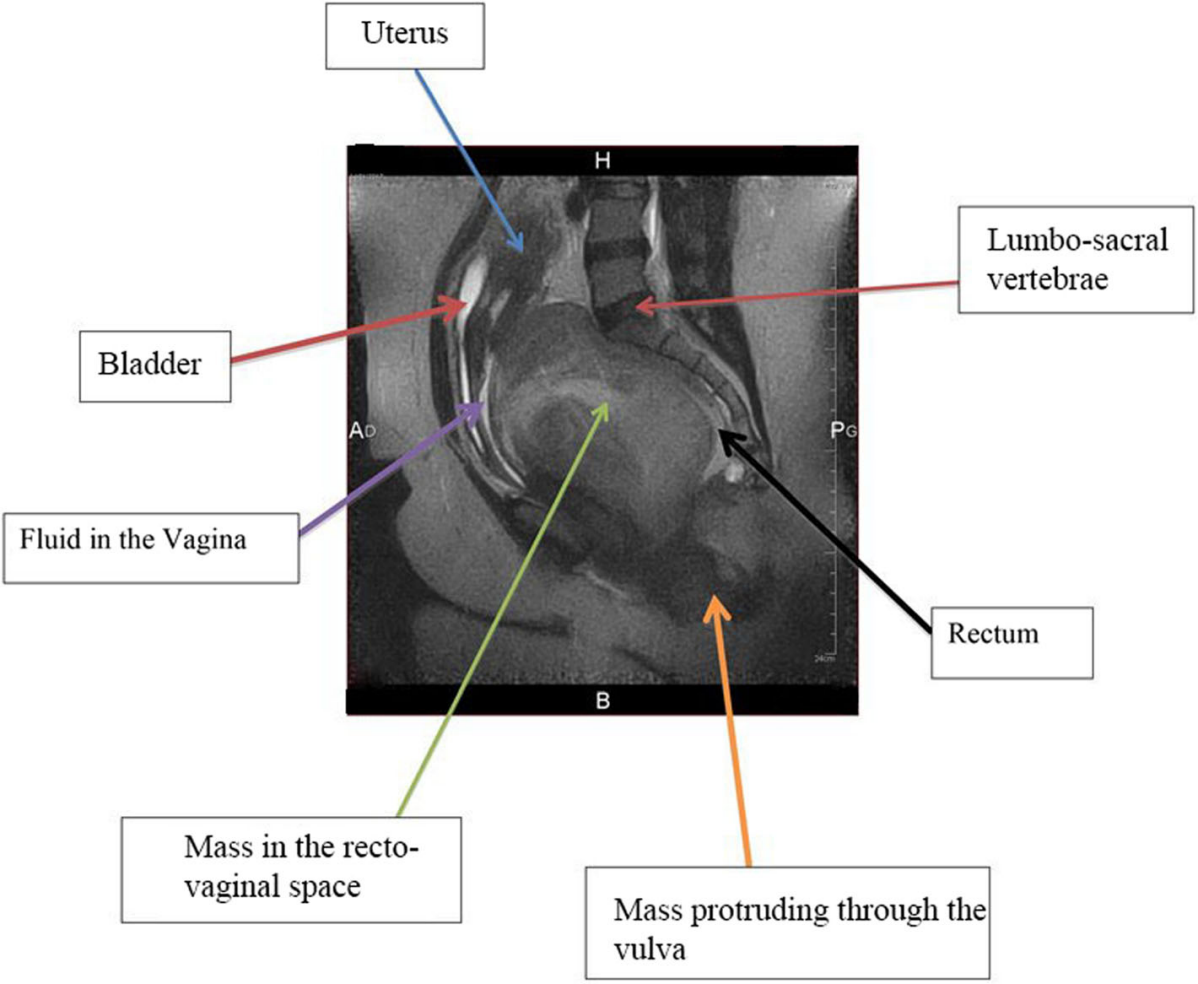
Magnetic Resonance Image (longitudinal view) of the pelvis and perineum showing pelvic mass, and fluid collection in the vagina. The uterus is deviated by the mass with secretions flowing through the cervix. The mass compresses the bladder anteriorly and the rectum posteriorly

**Fig. 3:**
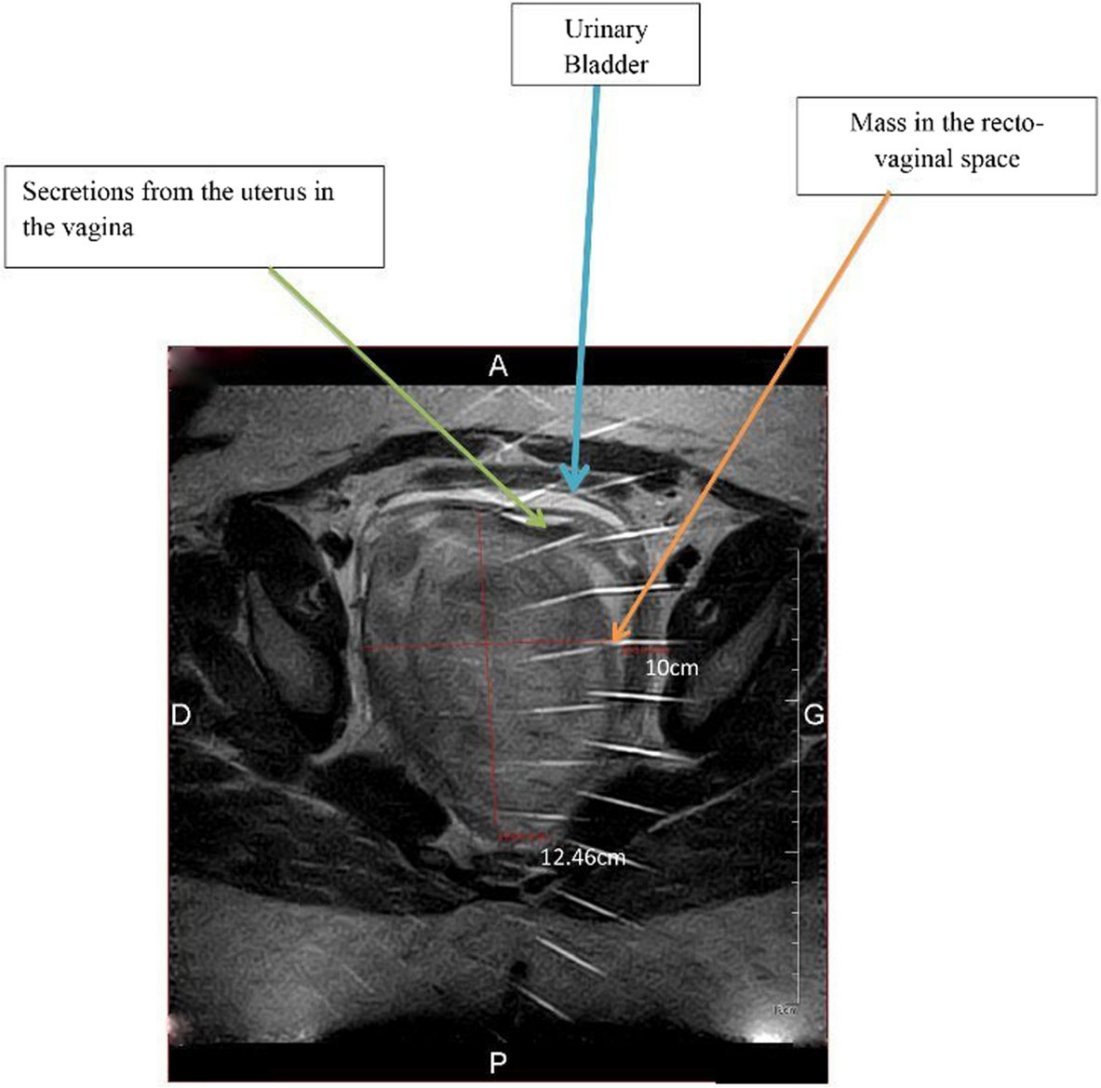
Magnetic resonance image (Cross-sectional view) of the pelvis and perineum. The uterus is not visualized in this section. The mass is seen with free fluid in the vagina

**Fig. 4:**
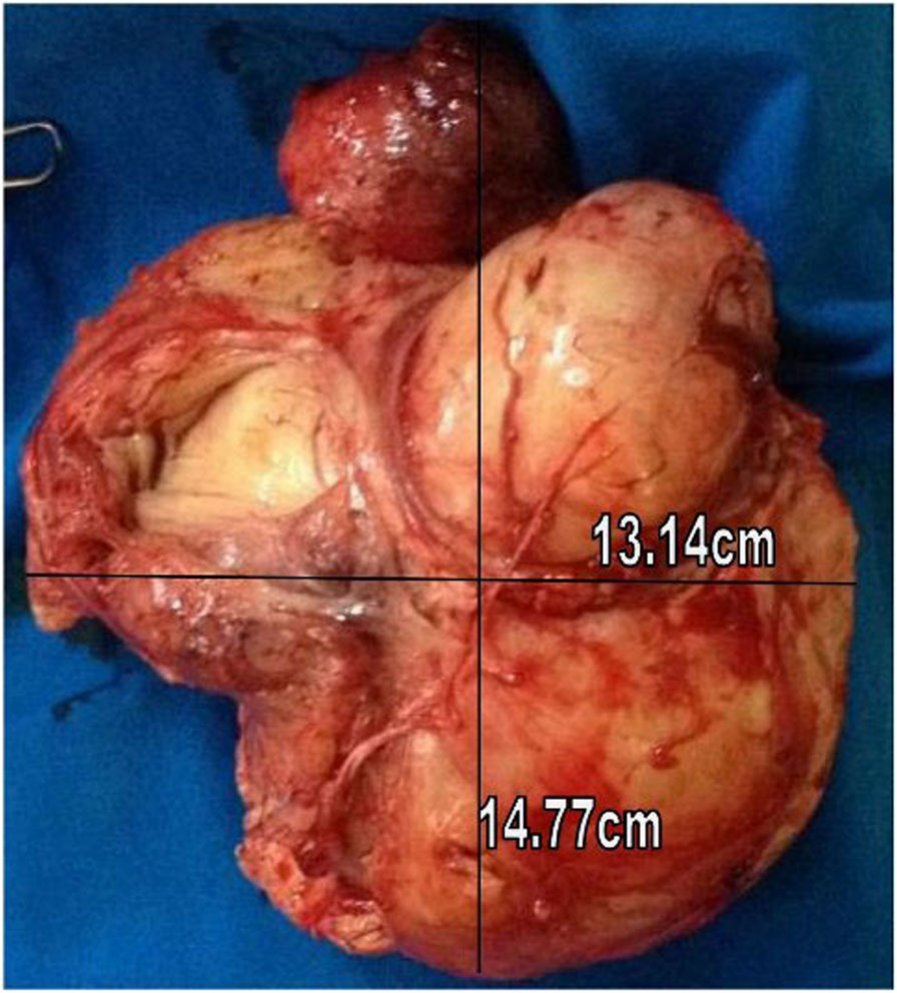
Vaginal tumour removed having whorled yellowish-white appearance characteristic of leiomyoma and measuring 13.14 cm × 14.77 cm

**Fig. 5:**
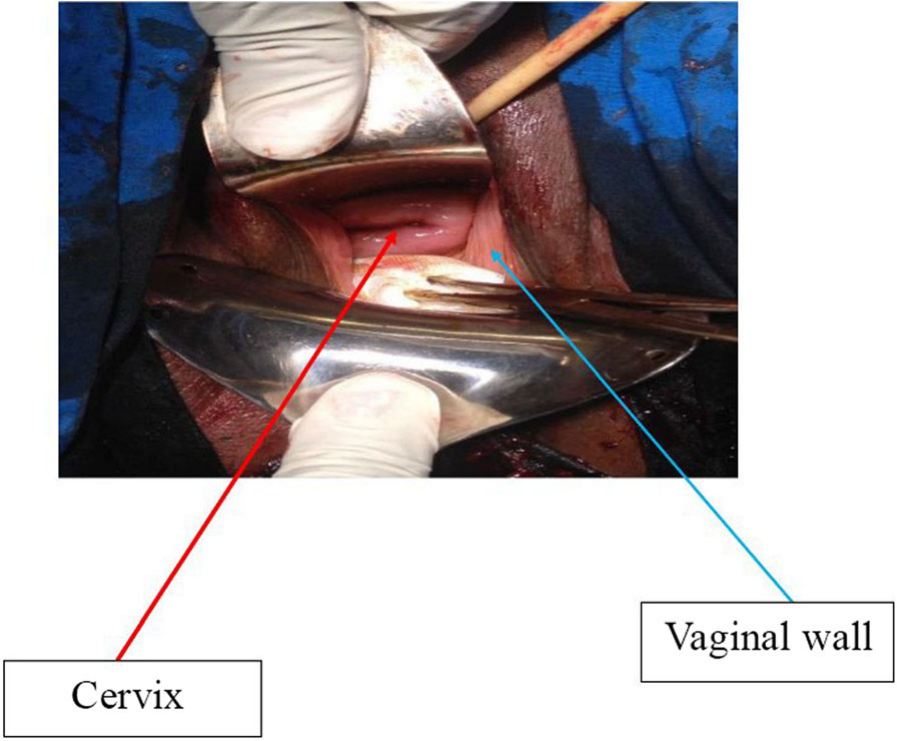
Vagina after removal of tumour. The uterine cervix is macroscopically normal

A 36-year-old G3P3002 sexually active Cameroonian married woman came to our department complaining of dysuria, dyspareunia, cessation of sexual intercourse and offensive foul-smelling vaginal discharge for a period of 6 months. She had noticed a growth bearing down on her vagina 3 years earlier and the growth rapidly increased in size in the last 6 months. On physical examination, she looked well; her blood pressure was 130/80 mmHg, pulse 84 beats/min. and temperature of 37.4 °C. She had a non-tender tumour that was rubbery in consistency and occluding the vagina thereby impeding access to the uterine cervix. On digital rectal examination, the tumour was impinging on and compressing both the bladder and the rectum (Fig. 1); allowing for micturition only in the erect position. Blood levels of tumour markers showed the following: CA 125; 12 IU/mL (normal value< 35 IU/mL), CA 19.9; 5.4 IU/mL (normal value < 37 IU/mL) and CA 15.3; 1.2 IU/mL (normal value< 31.3 IU/mL). Furthermore, hemoglobin level was 10.8 g/dL, hematocrit level 32.5%; aspartate aminotransferase (AST) 18.4 IU/L (normal value< 46 IU/L), alanine aminotransferase (ALT); 10.3 IU/L (normal value< 49 IU/L), blood urea nitrogen (BUN); 0.25 g/L (normal value; 0.15 to 0.45 g/L), and creatinine level 9.5 mg/L (normal value; 6 to 13 mg/L). Besides, immunohistochemistry was not accessible in the DRH during the period of study therefore we could not measure the LDH levels. An abdominal ultrasonography showed a 60 mm × 40 mm hypoechogenic tumour in the upper part of the vagina and pelvis. Magnetic resonance imaging (MRI) of the abdomen and pelvis had earlier misdiagnosed the tumour; right posterior pelvic peritoneal tumour measuring (137 × 73 × 118 mm). However, a re-examination of the MRI images by an experienced radiologist correctly diagnosed a vaginal tumour (102.7 × 175.8 mm) bulging through the posterior fornix and pushing up the pouch of Douglas and compressing the bladder and rectum, which is suggestive of vaginal leiomyoma (Fig. 2 and Fig. 3). The tumour was projecting through the vulva. A biopsy and histopathology of the visible vaginal tumour confirmed leiomyoma. At first look laparoscopy, the uterus and adnexa were normal and both deviated to the right side of the pelvis. We excised the leiomyoma through the vaginal route by sharp and blunt dissection. We removed a whorled whitish lobular vaginal fibroid (131.4 × 147.7 mm) occupying the rectovaginal space. The fourchette provided attachment for the vaginal leiomyoma (Fig. 4). Vaginal closure was in two layers and blood loss was minimal. The uterine cervix was microscopically normal (Fig. 5), and post-operative histopathology confirmed vaginal leiomyoma.

## Discussion and conclusion

Leiomyomas are benign tumours of the female genital tract common among African-American and black women of reproductive age [6–8]. These tumours are common in the uterus and to some extent, in the cervix, the round ligament, uterosacral ligament, ovary, and inguinal canal, respectively [1].

### Prevalence of vaginal leiomyoma

Leiomyomas of the vagina are rare and there are 300 reported cases in the medical literature we reviewed [1, 3]. Vaginal leiomyomas are common in women between 35 to 50 years and more common among Caucasian women [3]. Vaginal leiomyomas are rare in most sub-Saharan African countries, as opposed to uterine leiomyomas. The reason for this difference is attributed to misdiagnosis, failure to seek medical attention and lack of correct reporting.

### Diagnosis of vaginal leiomyoma

These tumours usually occur as a single, well-circumscribed mass arising from the midline anterior wall, and less commonly from the anterior and lateral walls [9]. In our case, the tumour was not well circumscribed as described in the literature and was located in the posterior wall (recto-vaginal space), thereby decreasing the clinical likelihood of suspicion. Vaginal tumours are usually asymptomatic but may occasionally present symptoms ranging from lower abdominal pain, low back pain, vaginal bleeding, dyspareunia, urinary symptoms like frequency, dysuria or other features of vagina or urinary obstruction [2, 10]. Preoperative imaging and careful examination may help rule out a malignancy. However, this was not the case with the index case that underwent biopsy because tumour was accessible. Besides, others have reported abnormally high levels of CA 125, CA 19.9 and CA 15.3 in 19.7%, 6.6% and 5.1% of patients, respectively [11] but we did not find similar results in the index case. Furthermore, elevated levels of lactate dehydrogenase LDH-A and LDH-D were reported in patients with uterine leiomyosarcoma compared with those with uterine leiomyomas [12]. In addition, other studies have suggested that, the assessment of LDH isoenzymes may be relevant in the preoperative diagnosis of uterine sarcoma [13]. However, LDH assessment was not accessible to the index case because of lack of facilities to carry out immunohistochemistry at the time of study. We used ultrasonography and MRI in diagnosing vaginal tumour. At the DRH, magnetic resonance imaging (MRI) is meant for suspected malignancies because it is not accessible to most patients due to the cost (about $250). In MRI, the tumour appears as a well-demarcated solid mass of low signal intensity in T1 and T2 weighted images with homogenous contrast enhancement while leiomyosarcomas and other vaginal malignancies show characteristic high T2 signal intensity with irregular and heterogeneous areas of necrosis or haemorrhage [9, 14, 15]. The use of MRI is especially useful when fibroids are growing rapidly, have poor delineation on ultrasound and when there is suspicion of malignancy [16]. MRI is accurate in diagnosing a leiomyoma with a sensitivity of 88–93% and a specificity of 66–91% [6].

There is a reported case of necrotizing ruptured leiomyoma mimicking a malignant neoplasm that was clarified by MRI. The mass was 70 mm × 50 mm at the distal end of the left anterior aspect of the vagina displacing the urethra anteriorly. It has low signal intensity on T1-weighted image, high signal intensity on T2 weighted image and irregular enhancement on post-contrast-enhanced image as if it were a malignant tumour [4].

There is an estimated 2 to 5% error margin in radiological examinations in the medical literature and this may increase to 40% in emergencies. We attribute these errors to complacency, poor communication, staff shortage, heavy workloads, a dearth of earlier reports for comparison, and unavailability of trained radiologists [17–20]. In the present context, there is no study in the Department of Radiology of the DRH to estimate radiologic accuracy. There are an estimated 140 radiology examinations per day in the Radiology Department of the DRH. Assuming a 2 to 5% margin of error [16–18] in radiologic diagnosis as reported previously, a misdiagnosis in diagnostic radiology may occur several times per day at the DRH.

### Management of Vaginal Leiomyomas

Surgical excision through the vaginal route has been the traditional approach for vaginal tumours but the abdominoperineal route is necessary for huge tumours [3, 19]. For the index case, we combined laparoscopic exploration of the abdominal cavity (internal genital organs) and per vaginal excision of the vaginal leiomyoma.

Some studies have reported the assessment of LDH isoenzymes levels by immunohistochemistry to differentiate between leiomyoma and leiomyosarcoma. However, because of the lack of immunohistochemistry facility and risk of misdiagnosis during MRI, frozen biopsy may be important to differentiate leiomyosarcoma from leiomyoma in our setting. Besides, other studies have reported that the overall accuracy of frozen section to determine the status of malignancy was 93.3% in patients with ovarian tumours [21]. Furthermore, another study have reported that Frozen section performed at the time of a total hysterectomy rendered the diagnosis of malignant mixed-müllerian tumour [22]. There are reports of laparoscopic ablation of a tumour with posterior colpotomy for the management of vaginal leiomyomas but this has not gained worldwide acceptance [23]. However, if vaginal leiomyoma is diagnosed before surgery, gonadotropin-releasing hormone analogues (GnRH-a) or selective progesterone receptor modulators (SPRM) to reduce tumour size or preoperative embolization to reduce intraoperative blood loss may be used [24]. Furthermore, there are reports of changes in sexuality and intimacy among patients after diagnosis and treatment of vaginal tumours [25]. This couple had also suspended sexual intercourse and therefore required psychological support.

### Limitations

There is a reported correlation between estrogen (ER) and epidermal growth factor (EGFR) receptors and vaginal leiomyoma [22] but we did not have the facilities to do immunohistochemistry. The couple could not get psychological support because of a lack of staff to offer such services.

In conclusion, posterior location of vaginal leiomyomas found in this case is a rare occurence. The diagnosis is based on careful examination and preoperative imaging (ultrasonography and MRI). However, definitive diagnosis is usually made intra-operatively. We combined laparoscopic exploration of the internal genital organs and per vaginal excision of the vaginal leiomyoma. Thus, we recommend frozen section to exclude leiomyosarcoma.

## Data Availability

Not applicable.

## Abbreviations

ALT: Alanine aminotransferase
AST: Aspartate aminotransferase
CA: Cancer antigen or carcinoma antigen
DRH: Douala Referral Hospital
EGFR: Epidermal growth factor
ER: Estrogen receptor
GnRH-a: Gonadotropin releasing hormone analog
LDH: Lactate dehydrogenase
MRI: Magnetic resonance imaging
SPRM: Selective progesterone receptor modulator
